# The Role of Neutrophils and Neutrophil Elastase in Pneumococcal Pneumonia

**DOI:** 10.3389/fcimb.2021.615959

**Published:** 2021-03-16

**Authors:** Hisanori Domon, Yutaka Terao

**Affiliations:** ^1^ Division of Microbiology and Infectious Diseases, Niigata University Graduate School of Medical and Dental Sciences, Niigata, Japan; ^2^ Research Center for Advanced Oral Science, Niigata University Graduate School of Medical and Dental Sciences, Niigata, Japan

**Keywords:** innate immunity, neutrophil, pneumonia, pneumolysin, neutrophil elastase, *Streptococcus pneumoniae*, virulence factor

## Abstract

*Streptococcus pneumoniae*, also known as pneumococcus, is a Gram-positive diplococcus and a major human pathogen. This bacterium is a leading cause of bacterial pneumonia, otitis media, meningitis, and septicemia, and is a major cause of morbidity and mortality worldwide. To date, studies on *S. pneumoniae* have mainly focused on the role of its virulence factors including toxins, cell surface proteins, and capsules. However, accumulating evidence indicates that in addition to these studies, knowledge of host factors and host-pathogen interactions is essential for understanding the pathogenesis of pneumococcal diseases. Recent studies have demonstrated that neutrophil accumulation, which is generally considered to play a critical role in host defense during bacterial infections, can significantly contribute to lung injury and immune subversion, leading to pneumococcal invasion of the bloodstream. Here, we review bacterial and host factors, focusing on the role of neutrophils and their elastase, which contribute to the progression of pneumococcal pneumonia.

## Introduction

Pneumonia is a common and serious infectious disease and has been a significant cause of morbidity and mortality worldwide, accounting for approximately three million deaths annually. The World Health Organization (WHO) placed lower respiratory infections as the fourth most common cause of death in 2016. Among a number of infectious agents, *Streptococcus pneumoniae*, also known as pneumococcus, is the most common cause of pneumonia in all age groups. In addition to localized infections such as pneumonia and otitis media, pneumococcus may cause invasive diseases, including meningitis and septicemia. Furthermore, an increase in antimicrobial resistance among pneumococci has raised concerns about the effectiveness of empiric antimicrobial therapy for pneumococcal pneumonia (Feldman, 2004; Ferrara, 2005; Nagai et al., 2019).

*S. pneumoniae* is a Gram-positive diplococcus that colonizes the mucosal surfaces of the human nasopharynx. Nasopharyngeal aerosolization of *S. pneumoniae* is considered to be the primary mode of population transmission. The molecular interaction of pneumococcal virulence factors and host proteins with respect to nasopharyngeal colonization has been thoroughly reviewed elsewhere (Kadioglu et al., 2008; Weiser et al., 2018). It has been reported that 18–92% of children are carriers of *S. pneumoniae* (Le Polain de Waroux et al., 2014); thus, they are considered the main reservoirs and transmission vectors of pneumonia (Smith et al., 2019). The aspiration of nasopharyngeal secretions leads to the invasion and propagation of *S. pneumoniae* in the lung parenchyma at the alveolar level, which leads to pulmonary infection (Liu et al., 2015). It has been reported that bacterial virulence factors directly damage human tissues or cause malfunctioning of the human immune system, resulting in an excessive inflammatory response. This excessive or inappropriate host inflammatory response is considered to result in the clinical syndrome of pneumonia. In this review, we discuss the bacterial and host factors that contribute to the progression of pneumococcal pneumonia, specifically focusing on the role of neutrophils and their elastase.

## Recognition of *S. pneumoniae* by the Innate Immune System of the Host

**Figure 1:**
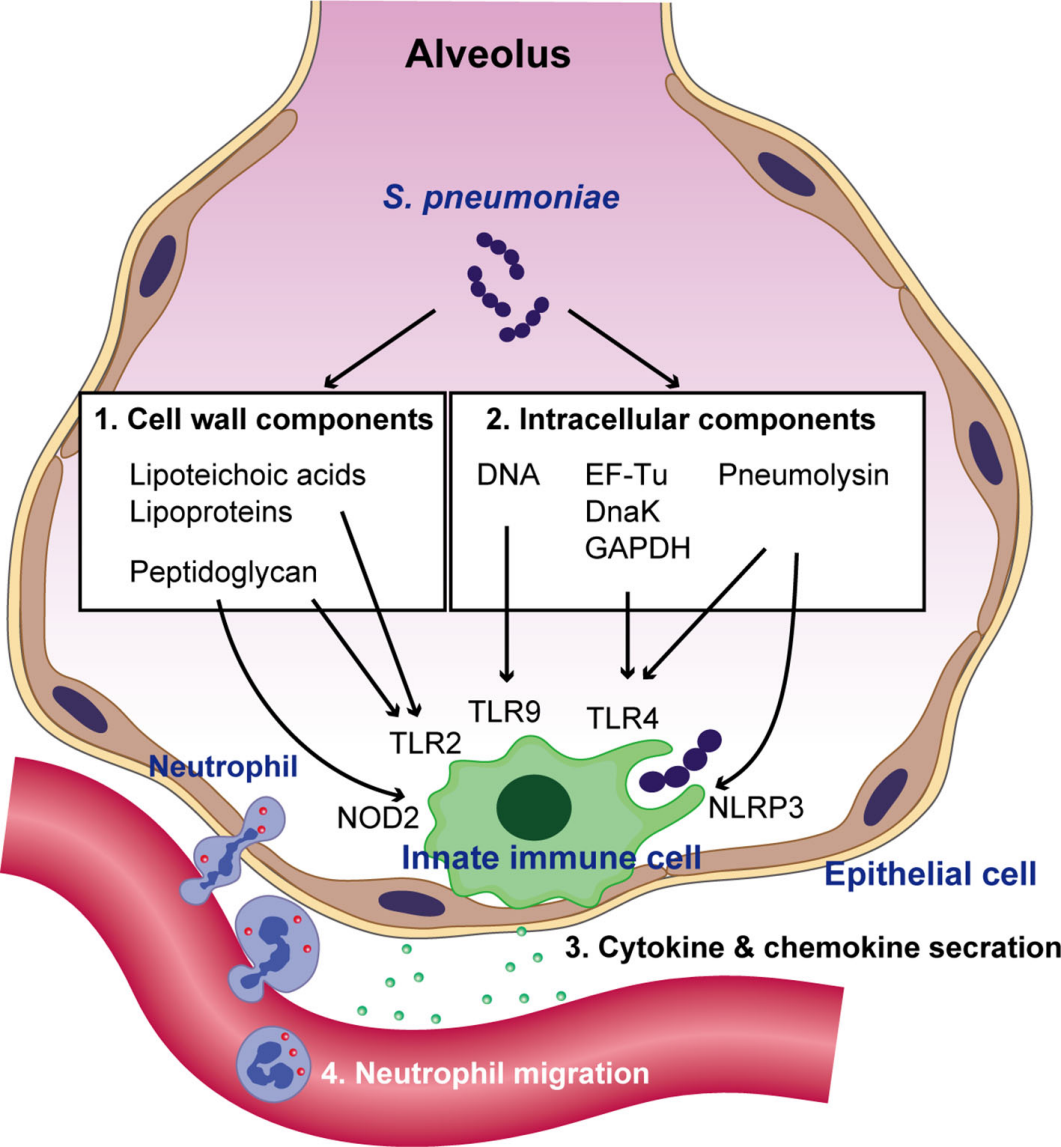
Recognition of *S. pneumoniae* by the innate immune system and neutrophil migration. Pneumococcal cell wall components such as lipoteichoic acids, lipoproteins, and peptidoglycan are recognized by TLR2. Following bacterial uptake by phagocytes and their degradation in phagosomes, pneumococcal peptidoglycan is recognized by NOD2. Pattern recognition receptors (PRRs) also sense pneumococcal intracellular molecules such as pneumolysin, genomic DNA, the chaperone protein DnaK, elongation factor Tu (EF-Tu), and glyceraldehyde-3-phosphate dehydrogenase (GAPDH). DNA is recognized by TLR9 within endosomes, whereas pneumolysin, DnaK, EF-Tu, and GAPDH are recognized by TLR4. Additionally, pneumolysin activates the NLRP3 inflammasome. The activation of PRR signaling leads to the transcriptional activation of cytokines and chemokines, which subsequently augments neutrophil migration.

Upon pneumococcal colonization or infection, the respiratory epithelium controls the bacterium through antimicrobial peptides, such as LL-37 and defensins (Bals and Hiemstra, 2004). However, *S. pneumoniae* can survive by removing its capsule from the surface (also see section 4) (Kietzman et al., 2016), which allows the organism to adhere to and invade the epithelium (Hammerschmidt et al., 2005). Pneumococcal interactions with other innate immune molecules, such as complements and surfactant protein-D, have been reviewed elsewhere (Kadioglu and Andrew, 2004). Following invasion of epithelial cells, the host innate immune system, which includes respiratory epithelial cells, alveolar macrophages, and dendritic cells, recognizes invading *S. pneumoniae* using pattern recognition receptors (PRRs) (Hartl et al., 2018). Different classes of PRRs include toll-like receptors (TLRs), nucleotide-binding oligomerization domain (NOD)-like receptors (NLRs), retinoic acid-inducible gene I-like receptors, and C-type lectin receptors (Takeuchi and Akira, 2010). These receptors are activated by conserved microbial molecules and bacterial virulence factors. Among the various TLRs, TLR2 recognizes several components of the pneumococcal cell wall, such as lipoteichoic acid, lipoproteins, and peptidoglycan (Yoshimura et al., 1999; Tomlinson et al., 2014), whereas TLR9 recognizes pneumococcal genomic DNA (Mogensen et al., 2006). Although TLR4 is known for its ability to detect lipopolysaccharide (LPS) from Gram-negative bacteria, it has been suggested that TLR4 might additionally recognize pneumolysin (Ply) (Malley et al., 2003), a pneumococcal pore-forming toxin. Additionally, we have demonstrated that pneumococcal cytosolic components, such as the chaperone protein DnaK, elongation factor Tu, and glyceraldehyde-3-phosphate dehydrogenase induce the production of proinflammatory cytokines *via* TLR4 (Nagai et al., 2018). NOD2 recognizes lysosome-digested peptidoglycan fragments of phagocytized *S. pneumoniae* (Davis et al., 2011). Additionally, Ply activates the NLRP3 inflammasome and promotes proinflammatory cytokine secretion by dendritic cells (McNeela et al., 2010). Intracellular signaling cascades triggered by PRRs lead to the transcriptional activation of inflammatory mediators, such as proinflammatory cytokines and chemokines. These mediators stimulate neighboring immune and non‐immune cells, activate the acute‐phase response, and recruit neutrophils (Koppe et al., 2012) (**Figure 1**).

## Neutrophil-Mediated Killing of *S. pneumoniae*

When infectious agents invade the respiratory tract, immune cells and epithelial cells secrete chemokines and cytokines, as described above, promoting neutrophil migration into the lung through the pulmonary capillary walls (Maas et al., 2018). Neutrophils phagocytose and kill infectious agents with the help of reactive oxygen species, antimicrobial proteins, and serine proteases (Teng et al., 2017). An *in vitro* study demonstrated that neutrophils degrade phagocytized *S. pneumoniae via* serine proteases such as neutrophil elastase (NE) and cathepsin G (CG), which are stored in azurophilic granules (Standish and Weiser, 2009). NE- and CG-deficient mice exhibit impaired antibacterial defense against *S. pneumoniae* and decrease murine survival without affecting neutrophil recruitment (Hahn et al., 2011). Furthermore, neutrophil depletion results in profound defects in the clearance of *S. pneumoniae* in a murine model of pneumonia (Garvy and Harmsen, 1996). This converging evidence indicates that phagocytic function and phagolysosomal degradation of bacteria by neutrophils are crucial strategies for controlling pneumococcal infection.

In addition to their phagocytic function, previous studies have reported that neutrophils release chromatin DNA decorated with granule-derived antimicrobial peptides and enzymes, including NE, CG, α-defensins, and myeloperoxidase (Brinkmann et al., 2004; Papayannopoulos, 2018). These chromatin structures are termed neutrophil extracellular traps (NETs), which degrade virulence factors and kill multiple microbial genera (Brinkmann et al., 2004). The trapping of microbes by NETs may provide several benefits, including reducing the spread of infection by concentrating host antimicrobial agents at infection sites. In bacterial pneumonia, animal and human studies have indicated that NETs are increased in alveolar spaces (Lefrançais et al., 2018; Mikacenic et al., 2018). Furthermore, an *in vitro* study demonstrated that NETs exhibit significant antibacterial activity against *S. pneumoniae* (Mori et al., 2012). These findings suggest the functional importance of NETs in pneumococcal pneumonia. However, higher concentrations of NETs have been reported to be associated with reduced hazards of clinical stability and increased mortality in pneumonia (Ebrahimi et al., 2018). In this context, excess NETs released by activated neutrophils have been implicated in promoting tissue damage, including sepsis (Czaikoski et al., 2016), and lung injury (Narasaraju et al., 2011). The mechanisms responsible for NET-induced tissue damage involve NET components such as NE and other proteases that induce cell death in multiple cell types (Yang et al., 1996; Hou et al., 2014; Grechowa et al., 2017; Daniel et al., 2019; Hiyoshi et al., 2019).

## Pneumococcal Virulence Factors Contribute to Evasion from Phagocytosis and Induce Neutrophil Death

A variety of pneumococcal virulence factors have been identified (Brooks and Mias, 2018; Feldman and Anderson, 2020). In the present review, we discuss the virulence factors associated with immune evasion.

The autolytic enzyme, autolysin, is known to be responsible for the characteristic autolytic behavior associated with pneumococci. The major autolysin of *S. pneumoniae* is *N*-acetylmuramyl-_L_-alanine amidase (LytA), which breaks down peptidoglycan (Höltje and Tomasz, 1976). Although the exact *in vivo* function of autolysis in pneumococcal pathogenesis is unclear, animal studies have demonstrated that pneumococcal strains deficient in LytA are less virulent than wild-type pneumococci (Berry et al., 1989a; Hirst et al., 2008). Recently, Kietzman et al. identified a novel physiological function of LytA. This enzyme was shown to drive rapid capsule shedding in response to antimicrobial peptides in the initial phases of infection (Kietzman et al., 2016). This response increases bacterial resistance to peptides, as well as invasion of the alveolar epithelium. LytA may also contribute to pneumococcal pathogenesis by catalyzing the release of the intracellular toxin Ply (Martner et al., 2008; Domon et al., 2016; Domon et al., 2018a), cell wall degradation products (Tuomanen et al., 1985), and cytosolic proteins (Nagai et al., 2018), which induce immune responses. Additionally, fragments from autolyzed bacteria inhibit phagocytosis of intact bacteria by peripheral blood mononuclear cells (Martner et al., 2009).

Ply is a potent intracellular toxin possessing multiple functions that augment pneumococcal virulence. Ply-deficient mutant strains of *S. pneumoniae* showed a significant reduction in virulence related to both intranasal and systemic infection (Berry et al., 1989b). Ply toxicity is mainly associated with its ability to induce ring-shaped pores in cholesterol-containing membranes (Tilley et al., 2005). In this regard, Ply has cytotoxic effects on various cell types, including alveolar epithelial cells (Rubins et al., 1993), microvascular endothelial cells (Zysk et al., 2001), and monocytes (Hirst et al., 2002). Thus, the direct cytotoxicity of Ply towards lung tissue is considered to play a primary role in lung injury in pneumococcal pneumonia. However, several studies have demonstrated that *in vivo* lung injury could be due to inflammation and microvascular leakage caused by Ply, rather than its cytotoxic activity (Witzenrath et al., 2006; García-Suárez et al., 2007; Witzenrath et al., 2007). Although neutrophils are required for the clearance of *S. pneumoniae*, intranasal or intratracheal infection of mice with wild-type *S. pneumoniae* demonstrated an increased neutrophil recruitment, increased bacterial burden in the lungs, and a higher prevalence of bacteremia compared to infection with Ply-negative mutant strains (Rubins et al., 1995; Kadioglu et al., 2000). Therefore, the proinflammatory interactions between Ply and neutrophils are considered to play a role in the aggravation of pneumococcal pneumonia. Indeed, Ply is cytotoxic to neutrophils (Cockeran et al., 2001). We demonstrated that Ply induces neutrophil cell death through specific interactions with the P2X_7_ receptor; whereas Ply is less cytotoxic against P2X_7_ receptor-negative alveolar epithelial cells and macrophages. This suggests that neutrophils are the primary target cells of Ply (Domon et al., 2016). The subsequent leakage of NE from dead neutrophils disrupts the pulmonary epithelial barrier. Another study demonstrated that Ply induces NET formation, which contains high levels of NE (Nel et al., 2016). Several others have reported pneumococcal evasion of NETs. Pneumococcal surface protein A plays a role in the resistance to NET-mediated killing (Martinez et al., 2019). Meanwhile pneumococcal endonucleases, EndA and TatD, allow the bacterium to degrade the DNA scaffolds of NETs and escape, followed by the release of NE from the NETs (Beiter et al., 2006; Jhelum et al., 2018).

A comprehensive review of the capsule, including its regulation in pathogenesis, capsule synthesis, and the genetic basis for serotype differences, has been published elsewhere (Paton and Trappetti, 2019). Accordingly, current mini reviews mainly focus on immune evasion related to virulence factors. The capsule, which confers protection against phagocytosis, has been extensively studied in the context of pneumococcal virulence (Jonsson et al., 1985; Andre et al., 2017). The capsule impairs bacterial opsonization with C3b/iC3b by the classical and alternative complement pathways and also inhibits the conversion of C3b, which bound to the bacterial surface, to iC3b, thus resulting in a profound inhibition of opsonophagocytosis by neutrophils (Hyams et al., 2010). Additionally, the capsule plays a role in bacterial adherence, colonization of the nasopharynx, and entry into alveolar epithelial cells (Hammerschmidt et al., 2005). Although capsules protect *S. pneumoniae* against trapping by NETs (Wartha et al., 2007), it has recently been observed that capsules of virulent pneumococcal serotypes enhance the formation of NETs during pneumonia (Moorthy et al., 2016). Moreover, NETs and neutrophil activity in the lungs generally correspond to disease severity after pneumococcal infection (Moorthy et al., 2016).

## Leakage of NE Causes Acute Lung Injury During Pneumonia

Neutrophil serine proteases, including NE, CG, and proteinase 3, are critical for the effective functioning of neutrophils, and contribute to immune protection (Pham, 2006). Among these proteases, NE has been well studied in both basic and clinical research. Although NE is a protease that degrades elastin (Janoff and Scherer, 1968), the degradation of foreign organic molecules phagocytosed by neutrophils is considered its main function (Kawabata et al., 2002). NE degrades outer membrane protein localized on the surface of Gram-negative bacteria to exert antimicrobial effects (Belaaouaj et al., 2000). NE-deficient mice are more susceptible to sepsis and death following infection with Gram-negative *Klebsiella pneumoniae* and *Escherichia coli* (Belaaouaj et al., 1998). However, the role of NE in Gram-positive bacterial infections remains controversial. It has been reported that NE does not contribute to neutrophil-mediated killing of Gram-positive *Staphylococcus aureus* (Belaaouaj et al., 1998). Specifically, in *S. pneumoniae*, NE plays an important role in degrading pneumococcal cell wall-localized aminopeptidase N, and mediates opsonophagocytic killing by neutrophils (Standish and Weiser, 2009; Nganje et al., 2019). However, some pneumococcal strains exhibit resistance to extracellular NE-mediated killing (Van der Windt et al., 2012; Domon et al., 2016).

Despite its fundamental importance in innate immunity, excessive neutrophil activation causes the release of NE, which contributes to tissue damage (Fox et al., 2013; Kovtun et al., 2018). In general, NE exerts potent catalytic effects against a broad array of host extracellular matrix components, including elastin, proteoglycan, fibronectin, and several collagen types (Janusz and Doherty, 1991; Taylor et al., 2018). The cross-linking of collagen and elastin imparts stability and functionality to the lung extracellular matrix, which plays an important role in the formation of alveolar gas exchange units (Mižíková et al., 2015). Many studies have indicated that increased NE activity in the lung is involved in the pathogenesis of various lung diseases such as pneumonia, acute lung injury, exacerbated chronic obstructive pulmonary disease, and cystic fibrosis (Polverino et al., 2017). Indeed, it has been reported that NE-deficient mice are protected to a significantly greater extent from the development of emphysema than wild-type mice (Shapiro et al., 2003). Additionally, N-formyl-methionyl-leucyl-phenylalanine-induced neutrophil influx in alveolar spaces results in decreased lung elastin content and the development of emphysema in mice (Cavarra et al., 1996). Moreover, instillation of NE into the lungs results in the destruction of alveolar walls in animals (Campbell, 2000). One possible mechanism could be that NE cleaves E-cadherin in alveolar epithelial cells which interferes with cell-cell adhesion (Boxio et al., 2016). As for bacterial infection, patients with bacterial pneumonia exhibit increased levels of NE in bronchoalveolar lavage fluid (BALF) (Boutten et al., 1996; Wilkinson et al., 2012), which may result in excessive proteolytic damage and worse clinical outcomes. Generally, NE is inhibited by serum α_1_-antitrypsin. However, neutrophils also release matrix metalloproteinases (MMPs) that inactivate α_1_-antitrypsin (Michaelis et al., 1990). Thus, the proteinase inhibitory capacity is decreased in the BALF in patients with bacterial pneumonia compared to that of healthy controls (Abrams et al., 1984).

**Figure 2:**
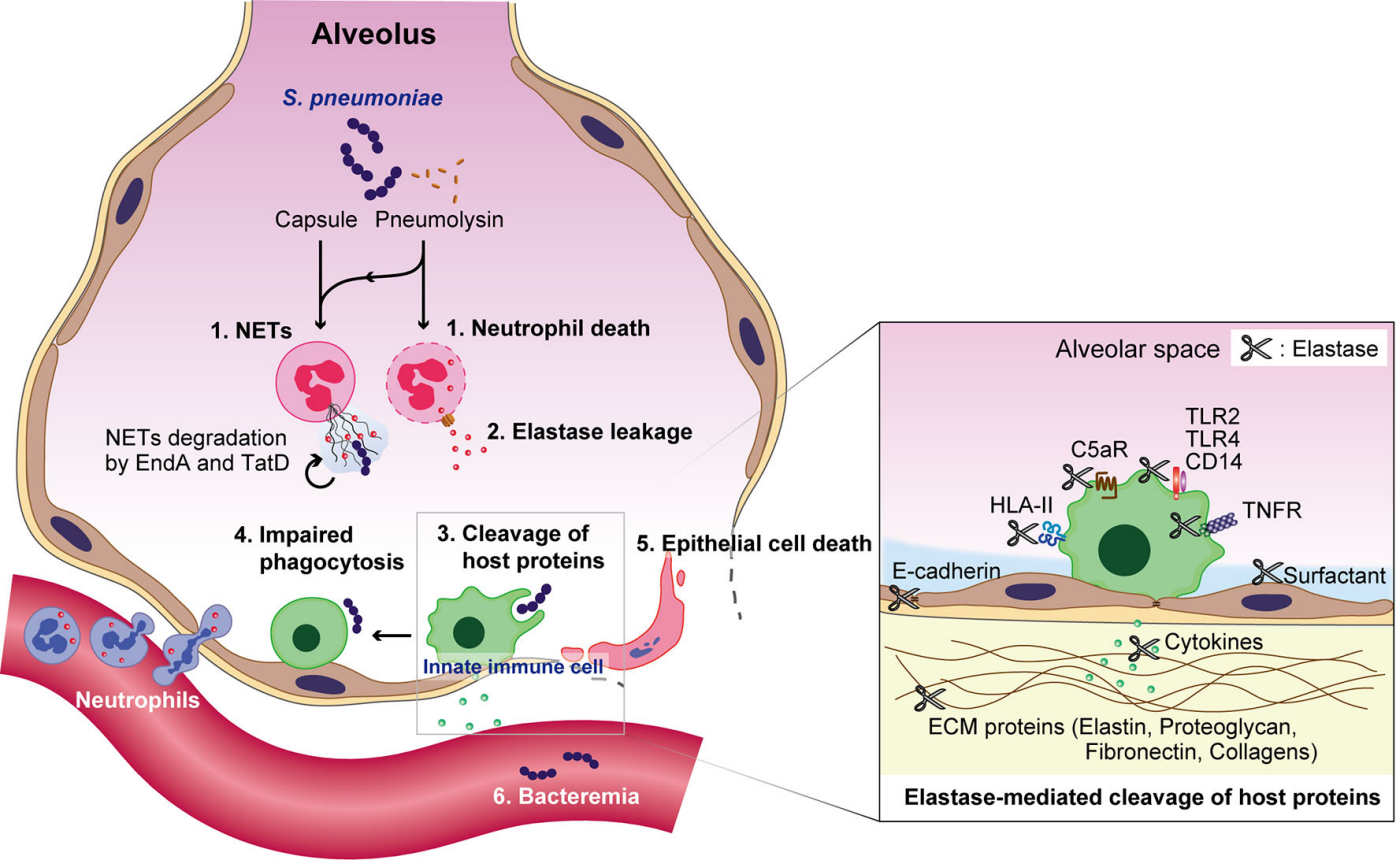
Overview of *S. pneumoniae*-induced immune subversion by the exploitation of neutrophils during pneumonia. Pneumococcal capsules and pneumolysin enhance the formation of neutrophil extracellular traps (NETs), which are subsequently degraded by the pneumococcal endonucleases EndA and TatD. Pneumolysin also exerts cytotoxicity against neutrophils. The subsequent leakage of neutrophil elastase induces the degradation of surfactant protein D, cell-cell adhesion molecule E-cadherin, and extracellular matrix components, such as elastin, proteoglycan, fibronectin, and several collagen types. Additionally, neutrophil elastase impairs the phagocytic activity of macrophages, induces the death of alveolar epithelial cells, and diminishes the pulmonary epithelial barrier. Furthermore, neutrophil elastase cleaves multiple cell surface proteins, such as toll-like receptor (TLR)2, TLR4, CD14, tumor necrosis factor receptor (TNFR), the C5a receptor (C5aR), and human leukocyte antigen class II (HLA-II); followed by the degradation of multiple cytokines and chemokines, such as interleukin (IL)-1β, IL-2, IL-6, IL-8, IL-12p40, IL-12p70, and tumor necrosis factor, which eventually disrupts the pulmonary immune defense.

In animal models, intratracheal pneumococcal infection causes acute lung injury, characterized by an increase in neutrophil accumulation and NE activity in the BALF (Yanagihara et al., 2007; Hagio et al., 2008). Subsequently, extracellular NE impairs the phagocytic activity of macrophages (Domon et al., 2016). Furthermore, NE cleaves extracellular matrix proteins, and proteins associated with the host immune response. In a murine model of bacterial pneumonia, NE, CG, and proteinase 3 cleave surfactant protein D, reducing the ability of the protein to promote bacterial aggregation (Hirche et al., 2004). NE also cleaves multiple cell surface receptors such as TLR2, TLR4, CD14, tumor necrosis factor receptor, and the C5a receptor, leading to an inhibition of downstream signaling (Wiedow and Meyer-Hoffert, 2005; Van den Berg et al., 2014; Domon et al., 2018b). Additionally, multiple cytokines and chemokines, such as interleukin (IL)-1β, IL-2, IL-6, IL-8, IL-12p40, IL-12p70, and tumor necrosis factor, are degraded and inactivated by NE (Wiedow and Meyer-Hoffert, 2005; Clancy et al., 2018; Domon et al., 2018b). Furthermore, we recently reported that NE cleaves human leukocyte antigen class II molecules in both cultured macrophages and *in vivo* mouse models, indicating that NE may disrupt antigen presentation and T-cell activation (Domon et al., 2021). In contrast, NE cleaves and activates MMP-9, which may also have a destructive role in lung diseases (Jackson et al., 2010). Collectively, these data imply that NE cleaves a variety of host immune proteins, induces lung injury, and may assist pneumococci in evading the immune system during pneumonia (**Figure 2**).

## Effect of NE Inhibitors on Bacterial Pneumonia

Multiple studies have investigated the potential role of various NE inhibitors, such as sivelestat (ONO-5046), AZD9668, EPI-hNE-4, KRP-109, and pre-elafin in different lung diseases (Polverino et al., 2017). In animal models of LPS- or chemical-induced non-infectious acute lung injury, symptoms have been observed to be ameliorated upon treatment with EPI-hNE-4 (Honoré et al., 2004), sivelestat (Sakamaki et al., 1996; Inoue et al., 2005; Iba et al., 2006), or pre-elafin (Vachon et al., 2002). Preclinical and clinical studies have also demonstrated the efficacy of sivelestat and AZD9668 in treating acute lung injury and bronchiectasis, respectively (Tamakuma et al., 2004; Inoue et al., 2006; Fujii et al., 2010; Aikawa et al., 2011; Stockley et al., 2013). In animal models of pneumococcal pneumonia, the administration of sivelestat resulted in higher survival rates and decreased bacterial counts in the blood (Yanagihara et al., 2007; Domon et al., 2018b), suggesting that NE-induced lung injury and immune subversion cause bacterial invasion of the bloodstream followed by death. Mice treated with KRP-109 showed lower neutrophil infiltration and inflammation than control mice, with no effects on viable pneumococcal numbers in the lungs (Yamada et al., 2011). These findings suggest that NE contributes, at least in part, to the pathogenesis of pneumococcal pneumonia. Although only a few studies have investigated the effects of NE inhibitors in patients with bacterial pneumonia, a retrospective study suggested that the early administration of sivelestat improves patient survival rate (Nakamura et al., 2008). Although this finding provides convincing evidence of NE-induced tissue destruction in pneumonia in humans, since neutrophils are the first phagocytic cells recruited to the lung infection site, further randomized controlled trials are required to examine the efficacy of NE inhibitors against bacterial pneumonia.

## Conclusion

Accumulating evidence indicates that both pneumococcal virulence factors and host proteases, including NE, are major mediators of lung injury during severe pneumococcal infections. Although the activation of PRRs in response to pneumococcal stimuli, followed by neutrophil infiltration, is key to the initiation of the innate immune response, this host defense strategy can be exploited by pneumococcus in lung tissues. *S. pneumoniae* targets infiltrated neutrophils and promotes the formation of NETs and cell lysis by utilizing Ply and other virulence factors, which in turn could increase the local NE concentration (Mori et al., 2012; Domon et al., 2016; Nel et al., 2016). Subsequently, elastase-induced proteolysis of extracellular matrix components (Taylor et al., 2018), cell-cell adhesion molecules (Boxio et al., 2016), and host immune molecules (Hirche et al., 2004; Wiedow and Meyer-Hoffert, 2005; Van den Berg et al., 2014; Clancy et al., 2018; Domon et al., 2018b) results in disruption of the alveolar epithelial barrier, which may allow pneumococci to invade the bloodstream. Additionally, several host proteases, including MMPs (Davey et al., 2011), CG, and proteinase 3 (Guyot et al., 2014), may contribute to lung injury. Thus, further basic research is still needed to understand the mechanisms of disease initiation, and to develop novel therapies for lung injury during bacterial pneumonia.

## Author Contributions

HD wrote the paper. HD and YT further developed and edited the manuscript. All authors contributed to the article and approved the submitted version.

## Funding

This work was supported by the Japan Society for the Promotion of Science (JSPS) KAKENHI Grant (JP20K09903, JP20K21671, and JP20H03858), the Takeda Science Foundation, and the Kobayashi International Scholarship Foundation.

## Conflict of Interest

The authors declare that the research was conducted in the absence of any commercial or financial relationships that could be construed as a potential conflict of interest.
